# Role of *GmFRI-1* in Regulating Soybean Nodule Formation Under Cold Stress

**DOI:** 10.3390/ijms26030879

**Published:** 2025-01-21

**Authors:** Hongcai Zhang, Lin He, Huiyun Li, Nengfu Tao, Tianda Chang, Dongmei Wang, Yichu Lu, Zhenying Li, Chunhai Mai, Xiaorui Zhao, Bingjie Niu, Junkui Ma, Lixiang Wang

**Affiliations:** Shanxi Houji Laboratory, College of Agriculture, Shanxi Agricultural University, Taigu 030801, China; zhanghongcai@sxau.edu.cn (H.Z.); z20213123@stu.sxau.edu.cn (L.H.); lihuiyun1@sxau.edu.cn (H.L.); taonengfu@sxau.edu.cn (N.T.); changtianda@sxau.edu.cn (T.C.); z20213104@stu.sxau.edu.cn (D.W.); luyichu1@sxau.edu.cn (Y.L.); lizhenying@sxau.edu.cn (Z.L.); maichunhai@sxau.edu.cn (C.M.); zhaoxiaorui@sxau.edu.cn (X.Z.); niubingjie1@sxau.edu.cn (B.N.)

**Keywords:** cold stress, *GmFRI-1*, nitrogen fixation, nod factor signaling pathway

## Abstract

Symbiotic nitrogen fixation, recognized as the most efficient nitrogen assimilation system in ecosystems, is essential for soybean growth, as nodulation provides critical nitrogen to host cells. Soybeans thrive in warm and moist environments. However, they are highly susceptible to low temperatures, which impede the formation and development of root nodules. The genetic basis and molecular mechanism underlying the inhibition of nodulation induced by low temperatures remain unclear. In this study, we conducted a comparative transcriptomic analysis of soybean roots inoculated with rhizobium at 1 DPI (Day Post Inoculation) under normal or cold treatments. We identified 39 up-regulated and 35 down-regulated genes associated with nodulation and nitrogen fixation. Notably, cold-responsive genes including three *FRI* (*Frigida*) family genes were identified among differentially expressed genes (DEGs). Further expression pattern analysis of *GmFRI-1* demonstrated it being significantly responsive to rhizobium inoculation and its highest expression in nodules. Further investigation revealed that overexpression of *GmFRI-1* led to an increase in the nodule number, while RNA interference (RNAi)-mediated gene editing of *GmFRI-1* suppressed nodule formation. Additionally, *GmFRI-1* overexpression may regulate soybean nodulation by modulating the expression of *GmNIN* (NODULE INCEPTION), *GmNSP1* (nodulation signaling pathway 1), and *GmHAP2-2* (histone- or haem-associated protein domain) in the nod factor signaling pathway. This study offers new insights into the genetic basis of nodulation regulation under cold stress in legumes and indicates that *GmFRI-1* may serve as a key regulator of nodule formation under cold stress.

## 1. Introduction

All plants require a specific temperatures range for their optimal growth and development, which varies among different species [1]. Cold stress occurs when an organism is exposed to temperatures beyond its normal range, and it can be classified into two types: hypothermia (occurring between 0 and 15 °C) and freezing (occurring below 0 °C) [2,3,4]. In recent years, research on plant responses to cold stress has achieved remarkable progress. When plants are subjected to low temperatures for extended periods, they undergo cold stress, resulting in aberrant alterations at multiple levels of cellular organization [5]. These alterations encompass modifications in membrane fluidity and damage [6], a decrease in the absorption of water and nutrients from the external environment leading to cellular starvation [7], alterations in the structure of proteins and nucleic acids, as well as a reduction in metabolic processes. Other effects include changes in gene expression [8], a decline in cellular respiration [9], an accumulation of osmotic pressure and cryoprotectants, and the production of reactive oxygen species [10,11,12].

Cold signal transduction in plants involves phytohormones, such as abscisic acid, auxins, and cytokinin, which mediate responses to cold stress by influencing the growth and tolerance mechanism [13,14]. Calcium acts as a secondary messenger, triggering various adaptive responses, including stomatal closure and alterations in gene expression [15]. Cold-regulated (*COR*) genes play crucial roles in protective responses, facilitating the production of antifreeze proteins and osmoprotectants like proline. Specifically, *COR47* and *COR15A/B* enhance cold tolerance by safeguarding against dehydration and stabilizing membranes during cold exposure [16,17,18,19,20]. Transcription factors, such as C-repeat Binding Factors (*CBF1*, *CBF2*, *CBF3*), activate the expression of cold-responsive genes, while Dehydration-Responsive Element-Binding (*DREB*) genes (*DREB1A/B/C*) regulate gene expression in response to cold and drought conditions [21,22]. Heat Shock Proteins (HSPs), including *HSP70*, *HSP90*, and *HSP100*, assist in protein folding and provide protection against stress [23]. Antifreeze proteins (AFPs) prevent the formation of ice crystal within plant tissues, while osmoprotectant synthesis genes, such as *P5CS*, are involved in proline synthesis, thereby stabilizing proteins and membranes [24,25]. Additionally, TPS contributes to trehalose production for osmotic protection, and sugar transporters (SWEET and STP) regulate osmotic balance under cold stress [26,27,28]. Reactive oxygen species (ROS) scavenging genes, including Superoxide Dismutase (SOD), catalase (CAT), and Ascorbate Peroxidase (APX), relieve cold stress by detoxifying superoxide radicals and reducing hydrogen peroxide levels [29,30,31,32]. Collectively, these genes enable plants to sense cold stress, activate protective mechanisms, and improve survival in low-temperature environments; examples of such genes include *COLD1* and *ICE1* [33,34].

The *FRIGIDA* (*FRI*) gene constitutes a crucial component in the regulation of cold response in plants [35]. *FRI* promotes flowering by facilitating the vernalization process, which necessitates prolonged exposure to low temperatures. This process is indispensable for plants to transition from a vegetative to a flowering state [36,37]. *FRI* functions by repressing the *FLOWERING LOCUS C* (*FLC*) gene, a significant flowering repressor. Under cold stress, *FRI* enhances the stability of *FLC* repressors, leading to *FLC* down-regulation and subsequent flowering [38,39]. Furthermore, *FRI* interacts with diverse signaling pathways involved in the cold response and plays a pivotal role in cold acclimation, aiding plants in adapting to low temperatures [35,40]. Additionally, *FRI* is implicated in epigenetic modifications that enable plants to retain the memory of cold exposure, thereby influencing gene expression patterns in subsequent generations [41,42,43]. It interacts with several proteins, including histone modifiers and transcription factors, which are essential for modulating the expression of cold-responsive genes [35,44]. Various alleles of *FRI* have been identified, demonstrating different responses to cold and influencing flowering time across diverse Arabidopsis ecotypes [45,46]. Studies indicate that the diversity of *FRI* alleles in natural populations of Arabidopsis reflects adaptation to local climatic conditions, underscoring its significance in plant evolution within low-temperature environments [47,48,49,50]. Manipulating *FRI* expression in transgenic plants has yielded insights into its role in cold tolerance and flowering [51,52]. Overexpression and knock out studies have demonstrated its dual function in regulating flowering and responding to cold [53].

Symbiotic nitrogen fixation is recognized as the predominant form of biological nitrogen fixation in the ecosystem [54]. In this process, rhizobia engage in a mutually beneficial relationship with legumes, resulting in the formation of root nodules or stem nodules in the roots or stems. This symbiotic association serves as a survival strategy for legumes within nitrogen-deficient environments [55]. The symbiotic interaction between rhizobia and legumes is characterized by mutual recognition and signal exchange, which involves the expression of numerous nodulation genes. Notably, nod factors (NFs) play a critical role in rhizobium recognition [56]. When the nitrogen levels in the soil are insufficient, legumes roots secrete compounds such as flavonoids and isoflavones. These compounds are detected by specific strains, which prompts the synthesis of lipogalactan oligosaccharides, known as NF. The NF receptors in the roots recognize NF, facilitating the interaction between rhizobia and the root epidermal cells of the host. This interaction induces the infection thread formation, cell dedifferentiation, and the subsequent division of cortical cells in the root system, ultimately leading to the development of a nodule that is capable of nitrogen fixation [56,57,58,59,60]. *ENOD* (early nodulation genes) genes are induced in the epidermis by rhizobia during early nodule formation, which is linked to nod factor perception and calcium spikes that facilitate rhizobial infection [61,62,63,64,65,66,67,68,69]. Additionally, NSP1 and NSP2 are essential for *ENOD* and CCaMK (calcium/calmodulin-dependent protein kinase), inducted by nod factors, while NIN is crucial for nodule formation and also influences *ENOD* expression, although its regulatory role may arise from the reduced inhibition of the nod factor response [70,71,72,73,74].

Soybean (*Glycine max*) is one of the most important legume plants, forming nodules to supply mainly nitrogen resources [75]. Soybean thrives in warm and humid climates. However, when exposed to temperatures as low as 15 °C, pod formation is completely inhibited, and nitrogen fixation can decline by as much as 45%, resulting in substantial losses in plant productivity [76,77]. Over the past two decades, significant advancements have been achieved in understanding the physiological responses of plants to low temperatures, encompassing photosynthesis, carbon metabolism, and nitrogen fixation [78,79]. Symbiotic nitrogen fixation (SNF) is especially vulnerable to low-temperature stress [80]. Lower temperatures inhibit root hair development in response to rhizobium, thereby affecting nodule formation, nodule development, nitrogen fixation, and assimilation [81]. Consequently, further investigation into the molecular mechanisms through which low temperatures impact nodulation in legumes is warranted.

In this study, we performed a comparative transcriptomic analysis of rhizobium-infected soybean roots under normal and cold treatment conditions. We identified the *GmFRI-1* gene, which is predominantly expressed in root nodules, and observed significant alterations in its expression due to cold stress. We employed overexpression (OE) and RNA interference (RNAi) to elucidate its role in nodule formation. The *GmFRI-1* positively regulates root hair deformation and nodule number, with the expression of marker genes associated with the nodulation factor (NF) signaling pathways showing substantial alterations, such as *GmNIN*, *GmNSP1*, and *GmHAP2-2*. This study pioneers in demonstrating the expression pattern and function of the renowned flowering and cold-responsive gene *GmFRI-1* in legume nodulation.

## 2. Results

### 2.1. Comparative Transcriptomic Analysis of DEGs in Soybean Root Inoculated with Rhizobium Under Normal or Cold Conditions

**Figure 1:**
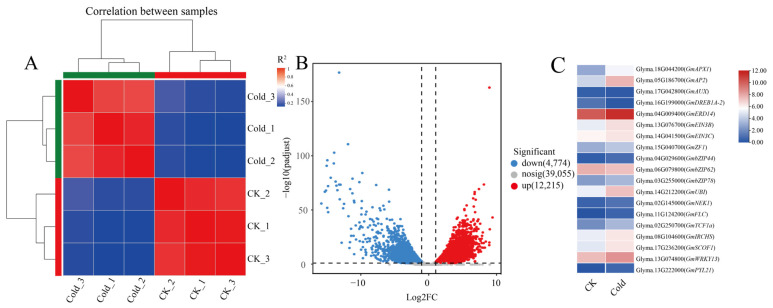
Transcriptomic analysis of soybean root infected by rhizobium under cold treatment or control at 1 DAI. (**A**) Principal component analysis of cold treatment or control soybean root samples infected by rhizobium. (**B**) Volcano map of DEGs between cold treated or control inoculated soybean root. (**C**) Differential expressed genes related to cold stress.

To investigate the effects of cold stress on the expression of soybean nodulation-related genes, soybean roots inoculated with rhizobium USDA110 were treated at 4 °C for 24 h, while the control group (CK) was maintained at room temperature. Illumina Novaseq™6000 sequencing generated approximately 285 million reads, with an average of 47.4 million reads per sample. The Q30 base ratio was 96%, and the GC content was approximately 44% (Table S1). Nearly 93% of the valid data were mapped to the reference genome (Glyma.Wm82.a4.v1), with approximately 83% of these reads being located at unique chromosomal positions (Table S2). Principal component analysis was conducted using FPKM values, resulting in a clear separation of samples into two distinct groups, along with their respective replicates (Figure 1A). To further verify the RNA-Seq data of differentially expressed genes (DEGs), we utilized quantitative real-time polymerase chain reaction (qRT-PCR) to validate the expression of six selected structural genes related to cold. The results of qRT-PCR confirmed the expression patterns of DEGs such as *GmAPX1*, *GmAUX*, and *GmDREB1A-2*, which were consistent with the Transcripts Per Million (TPM) values obtained from the RNA-seq analysis. This consistency supports the reliability of the gene expression data derived from the RNA-seq data (Figure S2).

As shown in the volcano plot, 12,215 up-regulated and 4774 down-regulated genes were identified through a comparative analysis of DEGs between the cold treatment group and the CK group (Figure 1B). The DEGs between the CK and treated groups were subjected to Gene Ontology (GO) annotation analysis, which indicated the DEGs enriched in protein-containing complexes (GO:0032991), cellular anatomical entities (GO:0110165), encompassed binding (GO:0005488), catalytic activity (GO:0003824), transcription regulator activity (GO:0140110), and ATP-dependent activity (GO:0140657). KEGG enrichment analyses demonstrated that the DEGs in response to cold treatment were mainly associated with the pathways related to Phenylpropanoid biosynthesis, Glycolysis/Gluconeogenesis and Starch, and sucrose metabolism (Figure S1A,B). Further analysis of these differential genes revealed that, in comparison to the control, there are 19 differential genes related to cold stress, such as *GmAPX1*, *GmDREB1A-2*, and *GmbZIP44* (Figure 1C); cold treatment resulted in a significant up-regulation of 39 genes associated with nodulation in soybean roots including *GmHAP2-1*, *GmERN1*, and *GmAUX1* (Figure S1C) and a significant down-regulation of 35 genes such as *GmLIN*, *GmRDN1*, and *GmCHC1* (Figure S1D). We also found that, compared to the control, cold stress significantly induced the expression of *FRI* and its homologous genes in soybean roots after rhizobium treatment, suggesting their potential involvement in cold stress-mediated nodulation regulation in soybeans.

### 2.2. Expression Patterns of Soybean FRIGIDA Family Genes in Response to Rhizobium Inoculation

**Figure 2:**
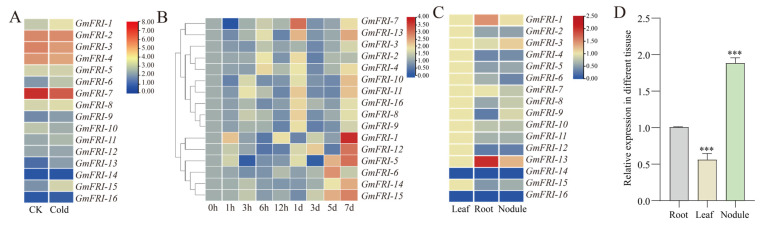
(**A**) Expression of *FRI* family members under cold treatment (4 °C) or control (room temperature). (**B**) Heat map shown the relative expression of soybean *FRI* family genes at different time points after inoculated with rhizobium USDA110. (**C**) The levels of relative expression of the *FRI* family genes within different tissues. (**D**) The relative expression level of *GmFRI-1* in different tissues at 28 DAI. The expression levels were normalized against the housekeeping gene of soybean *GmCYP2.* Student’s *t*-test was performed (*** *p* < 0.001, *n* = 10). Note: “*n*” represents the technical replicates of transgenic events used for statistics.

*FRI* family genes were found to be differential by cold treatment transcriptome analysis (Figure 2A). To check the expression patterns of soybean *FRIGIDA* family genes, soybean roots inoculated with rhizobium USDA110 were collected at 1, 3, 6, 12, and 24 HAI (Hours After Inoculation) and 3, 5, and 7 DAI (Days After Inoculation). The expression levels of 17 *FRIGIDA* family members were analyzed by qRT-PCR. As shown in Figure 2B, we found that most *FRIGIDA* family genes responded to rhizobium infection. The differing response patterns suggest the existence of functional divisions among *FRIGIDA* family members in soybean. *GmFRI-1* showed the most significant induction, which was observed on the seventh day. The digital expression level of *GmFRI-1* is relatively high in roots and nodules (Figure 2C). To confirm this, the expression levels of *GmFRI-1* in different tissues at 28 DAI were analyzed using qRT-PCR; compared to the root and leaf, the results showed that *GmFRI-1* was mainly expressed in the nodule (Figure 2D). This suggests that soybean may regulate cold stress-mediated nodulation through *GmFRI-1*.

### 2.3. Physiological Changes Related to Overexpressing GmFRI-1 (OE-GmFRI-1) Under Low Temperature Conditions

First, we constructed the 35S: *GmFRI-1* vector and obtained roots overexpressing *GmFRI-1* (OE-*GmFRI-1*), as confirmed by GFP fluorescence and qRT-PCR analysis (Figure S3A). The peroxidase (POD) activity of OE-*GmFRI-1* was remarkably elevated compared to the vector control (EV), both under room temperature and low-temperature conditions (Figure S3B,C). The proline content in the OE-*GmFRI-1* samples was also higher than that of the vector control (EV) at both low and room temperatures (Figure S3D,E). The malondialdehyde (MDA) content of *GmFRI-1* was significantly greater than that of the vector control (EV) at room temperature; however, no significant difference was observed under low temperature conditions (Figure S3F,G). The catalase (CAT) activity did not exhibit a significant disparity from that of the vector control (EV) (Figure S3H,I). These results shown that *GmFRI-1* significantly responds to cold stress in soybean.

### 2.4. GmFRI-1 Positively Controls Soybean Nodulation

**Figure 3:**
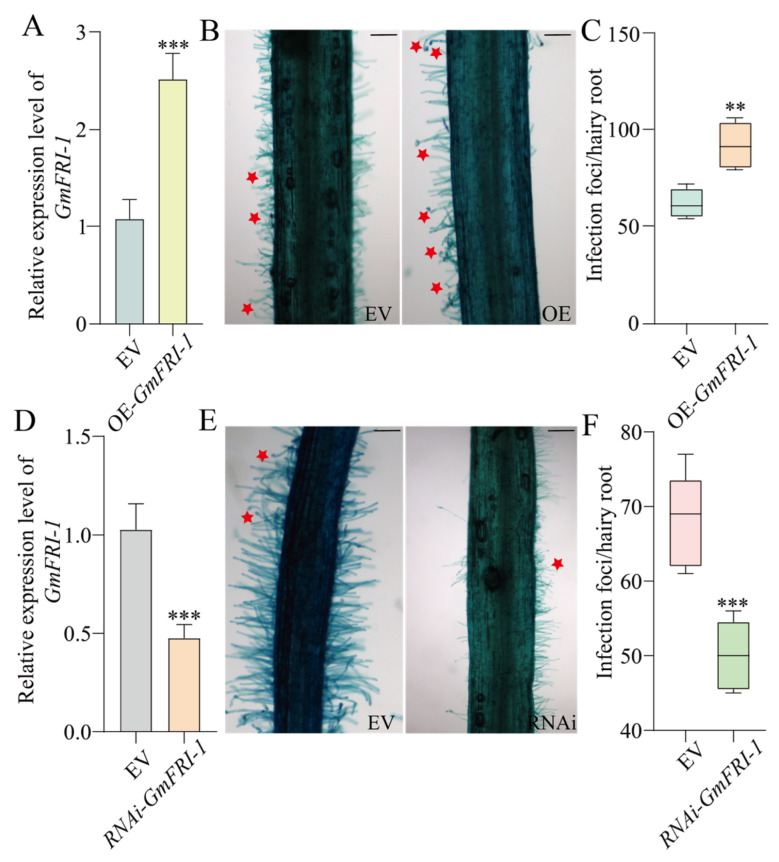
Root hair deformation status of *GmFRI-1* overexpression and knocking down soybean roots. (**A**) Expression level of transgenic hairy roots harboring empty vector and 35S: *GmFRI-1*. The expression levels were normalized against the housekeeping gene of soybean *GmCYP2*. Student’s *t*-test was performed (*** *p* < 0.001, *n* = 20). (**B**) At 7 DAI, 2 cm root segments of hairy roots overexpressing *GmFRI-1* or expressing EV below the root–hypocotyl junction were cut and stained with 1% (*w*/*v*) methylene blue. Deformed root hairs were counted (*n* = 20). Root hair deformation in transgenic roots harboring EV and 35S: *GmFRI-1* vector. Bar = 40 μm. The red star represents the typical root hair deformation beside it. (**C**) Quantification of deformed root hairs in the transgenic lines (*n* = 10 to 12). Values are averages ± SD from three independent experiments. Asterisks represent statistically significant differences. (*n* = 20, Student’s *t*-test; ** *p* < 0.01). (**D**) Expression level of transgenic hairy roots harboring empty vector and *GmFRI-1*-RNAi. The expression levels were normalized against the housekeeping gene of soybean *GmCYP2*. Student’s *t*-test was performed (*** *p*  <  0.001, *n* = 20). (**E**) Root hair deformation in transgenic roots harboring EV and RNAi-*GmFRI-1*, Bar = 40 μm. The red star represents the typical root hair deformation beside it. (**F**) Quantification of deformed root hairs in the transgenic root harboring EV and RNAi-*GmFRI-1* (*n* = 20). Values are averages ± SD from three independent experiments. Asterisks represent statistically significant differences. (*n* = 20, Student’s *t*-test; *** *p*  <  0.001). Note: “*n*” represents the technical replicates of transgenic events used for statistics.

**Figure 4:**
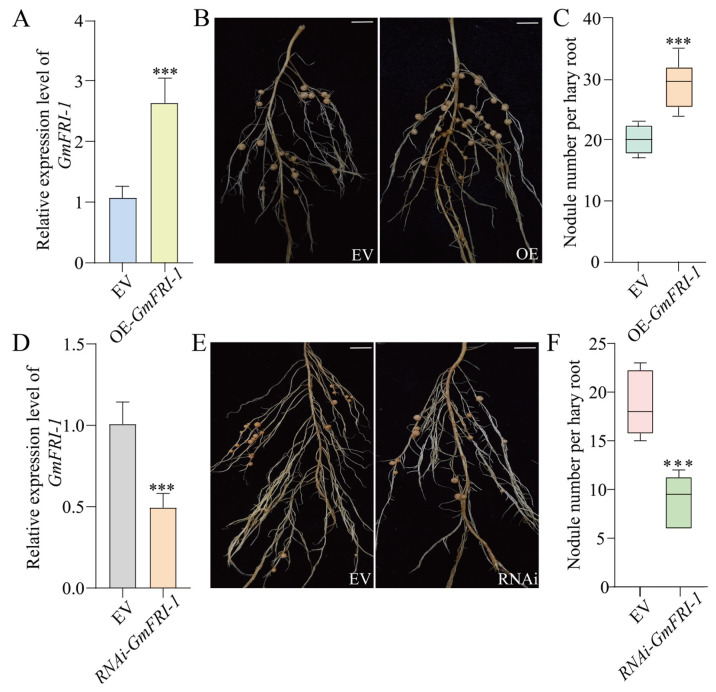
Nodulation status of *GmFRI-1* overexpression and knocking down soybean roots. (**A**) Expression level of transgenic hairy roots harboring empty vector and 35S: *GmFRI-1*. The expression levels were normalized against the housekeeping gene of soybean *GmCYP2*. Student’s *t*-test was performed (*** *p* < 0.001, *n* = 20). (**B**) Nodulation status of individual transgenic roots expressing EV and 35S: *GmFRI-1* at 28 DAI. Bar = 2 cm. (**C**) Quantification of deformed root hairs in the transgenic root harboring EV and *GmFRI-1*-RNAi (*n* = 20). Values are averages ± SD from three independent experiments. Asterisks represent statistically significant differences. (*n* = 20, Student’s *t*-test; *** *p*  <  0.001). (**D**) qRT-PCR analysis of transgenic hairy roots harboring empty vector and RNAi-*GmFRI-1*. The expression levels were normalized against the housekeeping gene of soybean *GmCYP2*. Student’s *t*-test was performed (*** *p* < 0.001, *n* = 15). (**E**) Nodule status of individual transgenic roots expressing empty vector and RNAi-*GmFRI-1* at 28 DAI. Bar = 2 cm. (**F**) Quantitative analysis of nodule number per hairy root carrying EV and RNAi-*GmFRI-1* at 28 DAI. Values are the mean ± SD. A total of 36 hairy roots were collected for each biological replicate (*n* = 12, Student’s *t*-test; *** *p* < 0.001). Note: “*n*” represents the technical replicates of transgenic events used for statistics.

To investigate the role of *GmFRI-1* in regulating soybean nodulation, we conducted overexpression and knocking down interference analyses of *GmFRI-1* using hairy root transformation system. The effects of OE-*GmFRI-1* on the early and late stages of nodulation were evaluated at 7 and 28 days after inoculation (DAI). We observed a marked increase in the number of deformed root hairs in OE-*GmFRI-1* hairy roots at 7 DAI compared to the vector control (EV) roots (Figure 3B,C). At 28 days, the average number of nodules per OE-*GmFRI-1* root was 29.1, compared to an average of 20 nodules in the EV control, reflecting a 45% increase in the nodule number following *GmFRI-1* overexpression. These findings suggest that *GmFRI-1* may positively regulate nodulation in soybean (Figure 4B,C).

To further investigate the role of internal *GmFRI-1* in soybean nodulation, we constructed a vector harboring RNAi-*GmFRI-1* (Figure 3D and Figure 4D). The analysis procedure for RNAi-*GmFRI-1* was identical to that described for OE-*GmFRI-1*. We observed a marked decrease in the number of deformed root hairs in RNAi-*GmFRI-1* hairy roots at 7 days after inoculation (DAI) compared to the empty vector (EV) control (Figure 3E,F). The average number of nodules in RNAi-*GmFRI-1* transgenic roots was 9, in contrast to the 18.6 nodules in the EV control roots, showing a 53% reduction in nodule number in *GmFRI-1* silenced roots (Figure 4E,F). In conclusion, these findings suggest that *GmFRI-1* positively regulates soybean nodulation.

### 2.5. GmFRI-1 Controls Soybean Nodulation Through Regulating Nodule Factor Signaling Pathway Genes

**Figure 5:**
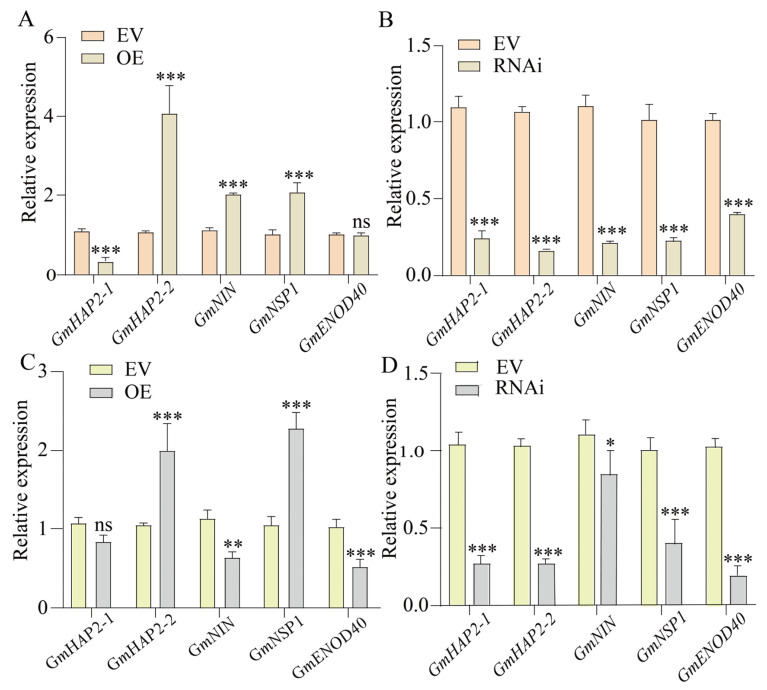
*GmFRI-1* affected the transcript levels of nodulation-related genes. (**A**) qRT-PCR analysis of *GmHAP2-1*, *GmHAP2-2*, *GmENOD40*, *GmNIN*, and *GmNSP1* in roots transformed with empty vector and *GmFRI-1*-OE at 1 DAI (*n* = 6). (**B**) qRT-PCR analysis of *GmHAP2-1*, *GmHAP2-2*, *GmENOD40*, *GmNIN*, and *GmNSP1* in roots transformed with empty vector and *GmFRI-1* knock out at 1 DAI (*n* = 6). (**C**) qRT-PCR analysis of *GmHAP2-1*, *GmHAP2-2*, *GmENOD40*, *GmNIN,* and *GmNSP1* in roots transformed with empty vector and *GmFRI-1*-OE at 5 DAI (*n* = 6). (**D**) qRT-PCR analysis of *GmHAP2-1*, *GmHAP2-2*, *GmENOD40*, *GmNIN,* and *GmNSP1* in roots transformed with empty vector and *GmFRI-1* knock out at 5 DAI (*n* = 6). The transcript amounts in each sample were normalized to those of *GmCYP2* (*n* = 6, Student’s *t*-test; * *p* < 0.05, ** *p* < 0.01, and *** *p* < 0.001; ns, no significance). Note: “*n*” represents the technical replicates of transgenic events used for statistics.

The alteration of *GmFRI-1* expression levels regulates the nodule number, which is primarily controlled by nod factor (NF) signaling pathways genes, including *GmHAP2-1*, *GmHAP2-2*, *GmNIN*, *GmENOD40*, and *GmNSP1*. We found at 1 DAI that the expression levels of *GmNSP1*, *GmNIN*, and *GmHAP2-2* were significantly elevated in the roots of OE-*GmFRI-1* (Figure 5A). In contrast, the expression of *GmNSP1, GmNIN*, and *GmHAP2-2* was markedly reduced in the RNAi-*GmFRI-1* roots (Figure 5B), with several other genes also showing significant reductions. As illustrated in Figure 5, the expression levels of *GmNSP1* and *GmHAP2-2* were significantly elevated in the roots of OE-*GmFRI-1* (Figure 5C). In contrast, the expression of *GmNSP1* and *GmHAP2-2* was markedly reduced in the root systems of the latter (Figure 5D), with several other genes also showing significant reductions. Therefore, it can be speculated that the *GmFRI-1* may influence soybean nodulation by regulating NF signaling pathway genes.

## 3. Discussion

Soybeans are well suited for cultivation in temperate regions where high nodulation and efficient nitrogen fixation occur [82]. However, low temperatures adversely impact the growth and development of soybeans. Cold stress can cause a reduction in pollen density, which in turn leads to fewer pods and a significant decrease in seed yield. Low temperatures exert an impact on every growth stage of soybeans, yet they are particularly detrimental to nodulation [83,84,85,86]. There have been only a limited number of studies regarding cold stress-mediated nodulation, and the genetic basis of symbiotic nitrogen fixation (SNF) in response to low temperatures in the nitrogen-fixing nodules of soybeans has yet to be unveiled. Our transcriptomic analysis of rhizobium-inoculated soybean roots under normal and cold conditions identified 39 up-regulated and 35 down-regulated nodulation-related genes, which suggests that cold stress affects classic nodulation pathways, thereby modifying soybean nodulation. Under low-temperature conditions, miRNA modules serve as a major regulator of miRNAs. MiRNAs target genes that can encode transcription factors, enzymes, and transport proteins, which are involved in a number of biological processes, including the activation/repression of downstream genes, redox, and the replication of organic/inorganic molecules related to nitrogen-fixing enzyme activity and rhizobium function [85]. For instance, Lotus *miR171* and *miR397* modulate rhizobium infection by targeting a transcription factor gene, the Nodulation Signaling Pathway, and a gene encoding a laccase copper protein, respectively [87,88]. Consequently, low temperatures reduce nitrogen fixation efficiency, emphasizing the importance of understanding the molecular mechanisms through which low temperatures influence the symbiotic relationship between soybeans and rhizobium. In this study, we identified cold-induced gene expression alteration in soybean nodulation through transcriptome analysis; among the differentially expressed genes, we discovered that *GmFRI-1* was up-regulated, with the highest expression observed at 7 DAI, as confirmed by qPCR analysis (Figure 2B), which indicates its putative role in cold-mediated soybean nodulation.

*FRIGIDA* (*FRI*) is a significant regulator of flowering time in Arabidopsis [89]. Its local expression in the phloem and leaves activates the target gene *FLC*, thereby delaying flowering time in these plants [90,91]. *FRI* proteins contain two convoluted helical motif structural domains and function as scaffolding proteins, having the ability to interact with a variety of proteins such as *SUF4*, *FLX*, *FES1*, *UBC1*, and *CBP20*. This interaction forms a complex with transcriptional activation capabilities, whereby the specific structural domain can enhance the expression of *FLC*, which is crucial for regulating the transcriptional level of *FLC* [57,58,92,93,94]. The expression pattern of legume *FRIGIDA* in relation to nodulation has yet to be thoroughly investigated. In this study, we discovered that *GmFRI-1* is predominantly expressed in nodules (Figure 2D), and most of the *FRI* family members identified in our transcriptome data were induced by cold treatment (Figure 2A). In Arabidopsis, grafting and genetic experiments have revealed that the local expression of *FRI* in the roots may generate a mobile signal that is transmitted to the shoot and antagonizes the FT signal, thereby delaying flowering [90]. Further investigation into the relationship of legume *FRIGIDA* among nitrogen, nodulation, and flowering will contribute to the elucidation of the underlying mechanisms of plant *FRIGIDA*.

Cold stress significantly affected the formation and function of soybean root nodules. Nodule formation, a critical process for nitrogen fixation, is negatively affected by low temperatures, resulting in a reduction in nodule number, size, and overall functionality [95,96,97]. Cold stress has the ability disrupt the signaling pathways involved in nodule development, which are crucial for both root and nodule growth [87,88]. In this study, we found that at 5 DAI, the expression levels of *GmNSP1* and *GmHAP2-2* were significantly elevated in the roots of OE-*GmFRI-1* compared to the empty vector (EV) control (Figure 5C). In contrast, the expression of *GmNSP1* and *GmHAP2-2* was markedly reduced in the root systems (Figure 5D), with *GmNIN*, *GmENOD40*, and *GmNSP1* also showing significant reductions. Regardless of whether it was under normal temperature circumstances or low temperature conditions, the activity of POD and the proline content in the state of overexpressing *GmFRI-1* were markedly greater than those in the control group (Figure S3B–E). Compared to the control, the overexpression of *GmFRI-1* resulted in significantly increased root hair deformations at 7 DAI and enhanced root nodule formation at 28 DAI (Figure 3B,C and Figure 4B,C). Conversely, the silencing of *GmFRI-1* led to significantly decreased root hair deformations at 7 DAI and reduced root nodule formation at 28 DAI (Figure 3E,F and Figure 4E,F). Under cold conditions, the physiological and metabolic processes that support nodule development may be hindered, causing a decrease in symbiotic efficiency with nitrogen-fixing bacteria [85]. Further studies on nodule formation and the nitrogen fixation efficiency of *GmFRI-1* are necessary to elucidate the effects of cold stress on soybean nodulation under cold conditions.

*GmFRI-1* is believed to interact with various signaling pathways, including those related to hormone regulation, stress response, and metabolic processes. Further research elucidating the downstream target genes and interaction partners of *GmFRI-1* during cold exposure, thereby highlighting its role in modulating cellular responses to cold stress and soybean nodulation, is warranted. Soybean cold-production regions are mainly distributed in the northeast of China, Russia, and Canada. Cold stress imposes prominent negative influences on soybean nodulation, yield, and seed quality. Symbiotic nitrogen fixation plays a very important role in the growth and development of soybeans; the identification of genes that confer the cold tolerance of soybeans and nodulation is important for soybean breeding in cold regions. This study provides important insights into how soybeans and legume nodulation adapt to cold environments and inform breeding strategies to enhance cold tolerance in soybean cultivars.

## 4. Materials and Methods

### 4.1. Plant Materials and Growth Conditions

Soybean (*Glycine max [L.] Merril*) cv Williams 82 plants were cultivated in vermiculite within a greenhouse environment (16 h light/8 h dark; 25 °C; 50% relative humidity), following the method described by Wang et al. [98]. For all nodulation experiments, *Bradyrhizobium japonicum* USDA110 (OD_600_ = 0.08) was utilized for inoculation. For the collection of the RNA-seq samples, soybeans were transferred to a diluted rhizobium solution at the emergence of the first trifoliolate leaf, achieved by softly rinsing the seedlings in phosphate-buffer solution (PBS, pH 7.5) to eliminate vermiculite and its particles. Subsequently, seedlings were placed in a 4 °C incubator for 24 HAI and lateral roots were collected [85,99,100,101], their control CK was placed in a room temperature incubator for 24 HAI and lateral roots were collected, and the collected lateral roots were frozen with liquid nitrogen for subsequent transcriptome analysis. Three independent biological replicates were performed, with each treatment containing more than 6 soybean seedlings.

### 4.2. Transcriptomic Analysis

Total RNA was extracted following the protocol established by Maha Osman et al., 2023 [102] and using the Trizol Reagent kit from Qiagen (Valencia, CA, USA). The purity and concentration of RNA were evaluated by the ratios of OD260/280 and OD260/230 using the Nanodrop 2000 spectrophotometer from Thermo Fisher Scientific (Wilmington, NC, USA). The double-stranded cDNA was used for generating the final cDNA library; the quality of libraries was evaluated on the Illumina Novaseq 6000 platform (San Diego, CA, USA). The transcriptome data have been submitted to the National Center for Biotechnology Information under accession number CRA015000. Differential expression analysis between samples was conducted with DESeq2, considering |log_2_ ^(Fold Change)^| ≥ 1 and padj ≤ 0.05 as the criteria for selecting differentially expressed genes. TPM (Transcripts Per Million) was applied to verify the identified DEGs in the Materials and Methods. Gene function annotation was conducted based on the following databases: non-redundant nucleotide sequence (NT), non-redundant protein sequence (Nr), Swiss-Prot, clusters of orthologous groups for complete eukaryotic genomes (KOGs), Kyoto Encyclopedia of Genes and Genomes (KEGG), and Gene Ontology (GO).

### 4.3. Gene Expression Analysis by Quantitative Real-Time PCR

The selected DEGs were validated using qRT-PCR assays. First strand cDNA synthesis (NovoScript^®^ Plus All-in-one 1st Strand cDNA Synthesis SuperMix (gDNA Purge) (Novoprotein, Suzhou, China) and qPCR reaction (NovoStart SYBR qPCR SuperMix Plus (Low ROX Premixed) (Novoprotein) were conducted following the manufacturer’s instructions. *GmCYP2* was employed as the internal reference gene. The relative expression of these 6 genes was determined using the 2^−ΔΔCt^ method [103]. The primer sequences are provided in Supplementary Table S3.

### 4.4. Expression Analysis of Differentially Expressed Genes

Soybean roots, leaves, and nodules samples were collected at 28 DAI, and then the relative expression level of *GmFRI* family members were analyzed using RT-qPCR. For the rhizobium response study, soybean roots were collected at different time points: 0 h, 1 h, 3 h, 6 h, 12 h, 24 h, 72 h, 120 h, and 168 h. RT-qPCR was performed to evaluate the expression level at different time points; *GmCYP2* was utilized as an internal control [104]. RT-qPCR was conducted with a qPCR kit (ROCGENE, Beijing, China) and the CFX96™ real-time PCR detection system (Bio-Rad, Hercules, California, USA), with data processed according to the 2^−ΔΔCt^ method. Statistical analyses are represented in the graphs with asterisks indicating significance levels, (* *p* < 0.05, ** *p* < 0.01, and *** *p* < 0.001). PCR primer sequences for the studied genes were listed in Supplementary Table S3.

### 4.5. Measurement and Analysis of Associated Physiological Parameters

The *GmFRI-1* overexpression materials and the control were promptly placed into an incubator at 4 °C when inoculated with rhizobia. The control was placed at room temperature for one day before its lateral roots were collected. The POD activity, MDA content, Pro content, and CAT activity of the root system were measured. The POD activity was determined by the guaiacol method, the MDA content was measured by the thiobarbituric acid (TBA) method, the Pro content was measured by the ninhydrin method, and the CAT activity was measured by the hydrogen peroxide method [105].

### 4.6. Plasmid Construction and Genetic Transformation of Soybean Hairy Roots

*GmFRI-1* was amplified from soybean cDNA, followed by cloning into the pUBI-GFP-4×myc vector with the CaMV 35S promoter and Nos terminator to generate pUBI-*GmFRI-1*-4×myc plasmid. *GmFRI-1* CDS was ligated into the pK7WG2D-GFP vector to create RNAi-*GmFRI-1* plasmid. The primers used for gene cloning and plasmid construction of *GmFRI-1* are listed in Supplementary Table S3. After transforming the empty vector (EV; pUBI-GFP-4×myc, pK7WG2D-GFP), pUBI-*GmFRI*-1×myc and RNAi-*GmFRI-1* plasmids are transformed into Agrobacterium rhizogenes K599 by electroporation. Sterilized seeds of soybean were germinated on B5 medium for 5–7 days. After making incisions at the hypocotyls, explants were then immersed in a K599 suspension for 40 min at an OD_600_ = 0.5. Co-cultivation was conducted in the dark at 25 °C for 2–3 days. Subsequently, explants were transplanted into vermiculite for rooting. Transgenic hairy roots were assessed using GFP fluorescence using a specific light source after approximately 6 days of transplantation. Seedlings with fluorescent roots were considered positive and continued to grow in vermiculite. The transgenic hairy roots were immediately frozen in liquid nitrogen and stored at −80 °C after collection. Approximately 0.1 g of transgenic hairy roots was used for RNA extraction and quantitative real-time PCR analysis; EV, overexpressed *GmFRI-1,* or RNAi-*GmFRI-1* roots were then collected for further phenotype analysis.

### 4.7. Statistical Data Analysis

Student’s *t*-test was performed using GraphPad Prism version 8.0.0 to validate the differences in nodulation or the gene expression of the manuscript. * *p* < 0.05, ** *p* < 0.01, and *** *p* < 0.001.

## 5. Conclusions

In this study, we identified the *FRI* family through transcriptome analysis following cold treatment and discovered that *GmFRI-1* was most highly expressed 7 DAI, as well as in soybean rhizomes. Significantly, we observed that OE-*GmFRI-1* led to an increase in the number of soybean nodules. Conversely, RNAi-*GmFRI-1* inhibited soybean nodule formation. Furthermore, we demonstrated that variations in *GmFRI-1* expression had an impact on the expression of genes of the NF signaling pathway, which encompasses processes such as rhizobium infection and nodule formation. These research findings will serve as a solid foundation for a more profound comprehension of *GmFRI-1* and its role in the nodulation process. This study offers new insights for further investigation into the function of the *FRIGIDA* in legume nodulation and highlights the significance of *GmFRI-1* as a key gene in the regulation of legume symbiotic nodulation mediated by cold stress.

## Data Availability

The RNA-seq data produced in this study were submitted in to The transcriptome data have been submitted to the National Center for Biotechnology Information database, accession no: subCRA032379. Sequence data from this article can be found in the GenBank/EMBL or *Glycine max* Wm82.a4.v1 database.

## Supplementary Materials

The following supporting information can be downloaded at https://www.mdpi.com/article/10.3390/ijms26030879/s1.
