# Supplementary material for: Role of GmFRI-1 in Regulating Soybean Nodule Formation Under Cold Stress
Source: Int J Mol Sci. 2025 Jan 21;26(3):879. doi: 10.3390/ijms26030879 (PMC11816883; doi:10.3390/ijms26030879)
Supplement: Supplementary file 1 [file ijms-26-00879-s001.zip › ijms-3317201-supplementary.pdf]

Table S1 Statistics of RNA-Seq data

Sample	Raw_reads	Raw_bases	Clean_reads	Clean_Bases	Error%	Q20%	Q30%	GC%
CK_1	48204598	7278894298	47789094	7142199762	0.0118	98.82	96.42	44.82
CK_2	44719552	6752652352	44334194	6630329093	0.0118	98.79	96.35	44.78
CK_3	49643216	7496125616	49221166	7336118793	0.0119	98.8	96.33	45.13
Cold_1	48056382	7256513682	47643232	7072558229	0.012	98.73	96.15	44.75
Cold_2	45901656	6931150056	45510962	6788486805	0.012	98.7	96.06	44.63
Cold_3	47680760	7199794760	47301546	7063252084	0.0117	98.86	96.57	44.48

Table S2 Statistics on the distribution of reads in different regions of the reference genome

Sample	CDS	5'UTR	3'UTR	Introns	Intergenic	Total_mapped
CK_1	56082064.0(85.7%)	3814084.0(5.83%)	2893597.0(4.42%)	2138274.0(3.27%)	511660.0(0.78%)	44683806(93.5%)
CK_2	53581435.0(84.93%)	4195090.0(6.65%)	2673588.0(4.24%)	2105104.0(3.34%)	537214.0(0.85%)	42318493(95.45%)
CK_3	59492843.0(86.08%)	4006718.0(5.8%)	2916403.0(4.22%)	2160321.0(3.13%)	538889.0(0.78%)	47244738(95.98%)
Cold_1	52767031.0(82.72%)	4284388.0(6.72%)	2763699.0(4.33%)	3522971.0(5.52%)	453693.0(0.71%)	45038749(94.53%)
Cold_2	51819877.0(83.12%)	4487696.0(7.2%)	2904083.0(4.66%)	2724746.0(4.37%)	409431.0(0.66%)	43337474(95.22%)
Cold_3	52809700.0(82.39%)	1714893.0(2.68%)	7835452.0(12.22%)	1265202.0(1.97%)	475292.0(0.74%)	45373551(95.92%)

Note: Distribution of reads in different regions of the reference genome. (1) CDS: Coding region. (2) Intergenic: intergenic region. (3) Introns: intronic region. (4) 3'UTR, 5'UTR: Untranslated region at the 3' or 5' end of mRNA. (5) Total reads: Statistics of the sequences number after filtered sequences.

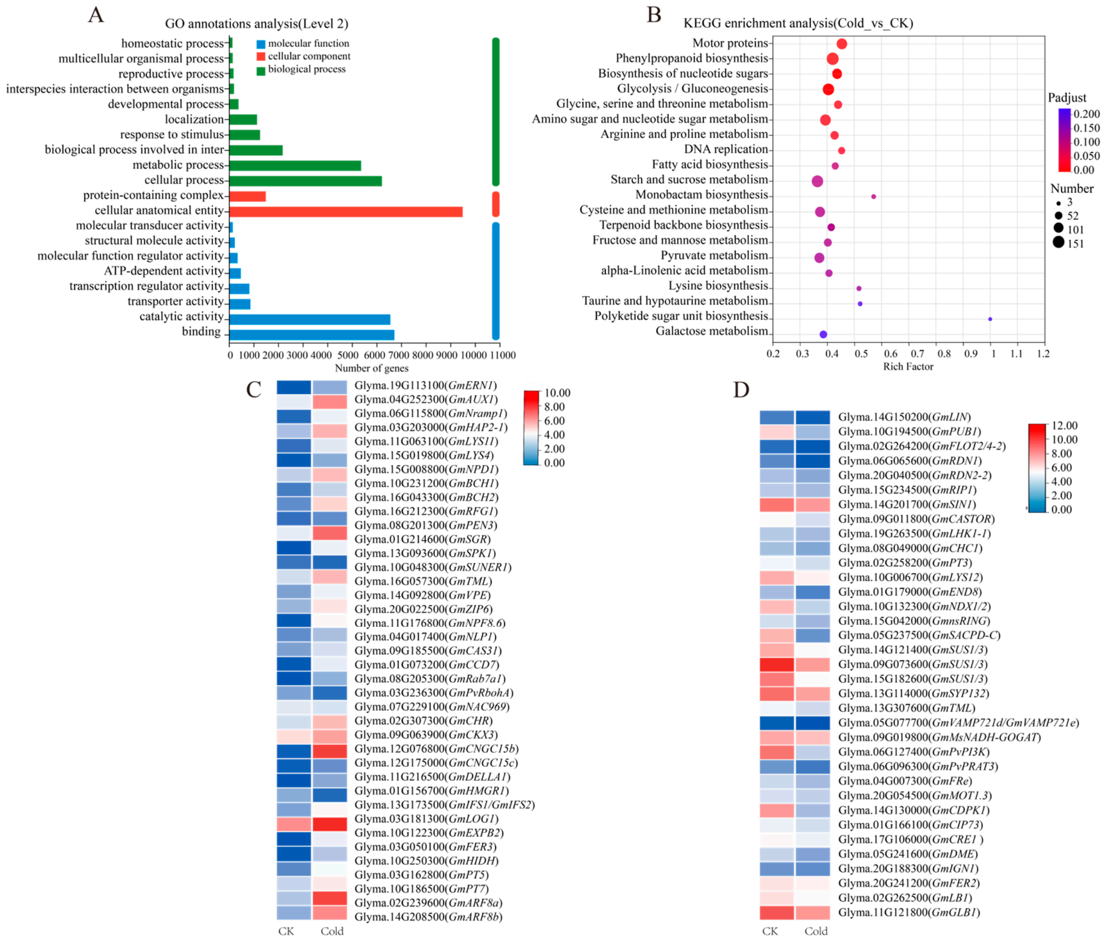

Supplementary Figure S1. Annotation of the DEGs and differentially expressed genes in nodulation. (**A**) GO annotation of DEGs. (**B**) KEGG pathway enrichment analysis DEGs. (**C**) Up-regulated genes associated with nodulation in response to cold stress. (**D**) Down-regulated genes associated with nodulation in response to cold stress.

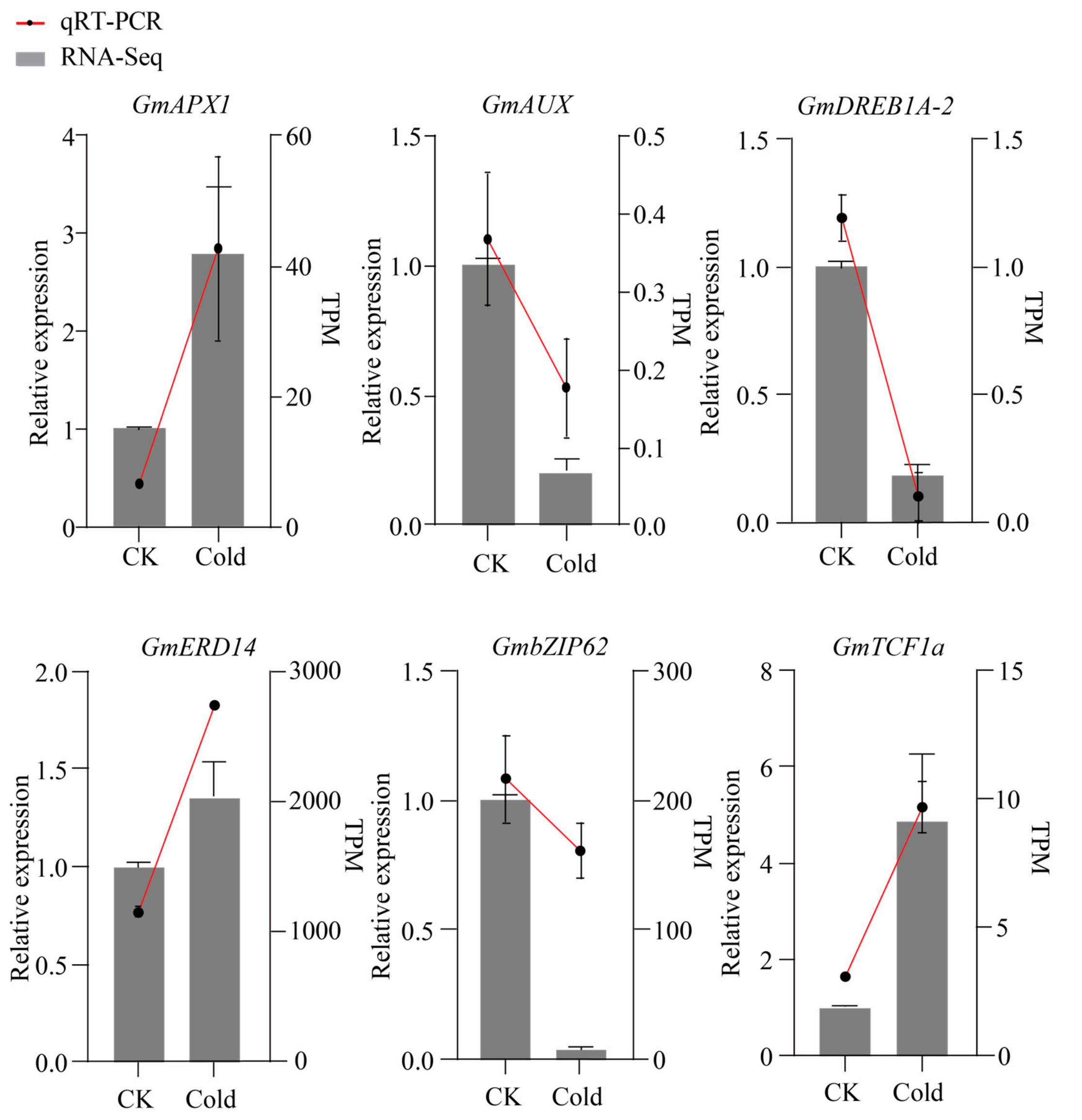

Supplementary Figure S2. Verify the expression levels of cold-related genes. qRT-PCR was used to analyze the relative expression levels of six cold-related DEGs in CK and cold-treated samples. The left y-axis represents the expression data of RT-qPCR (black histograms), while the right y-axis represents the relative gene expression levels measured by RNA-seq (red lines). *GmCYP2* was used as the reference gene for normalization. qRT-PCR only analyzed significant differences, and the values shown are SD ± mean (**p*<0.05, ***p*<0.01, and ****p*<0.001; ns, no significance).

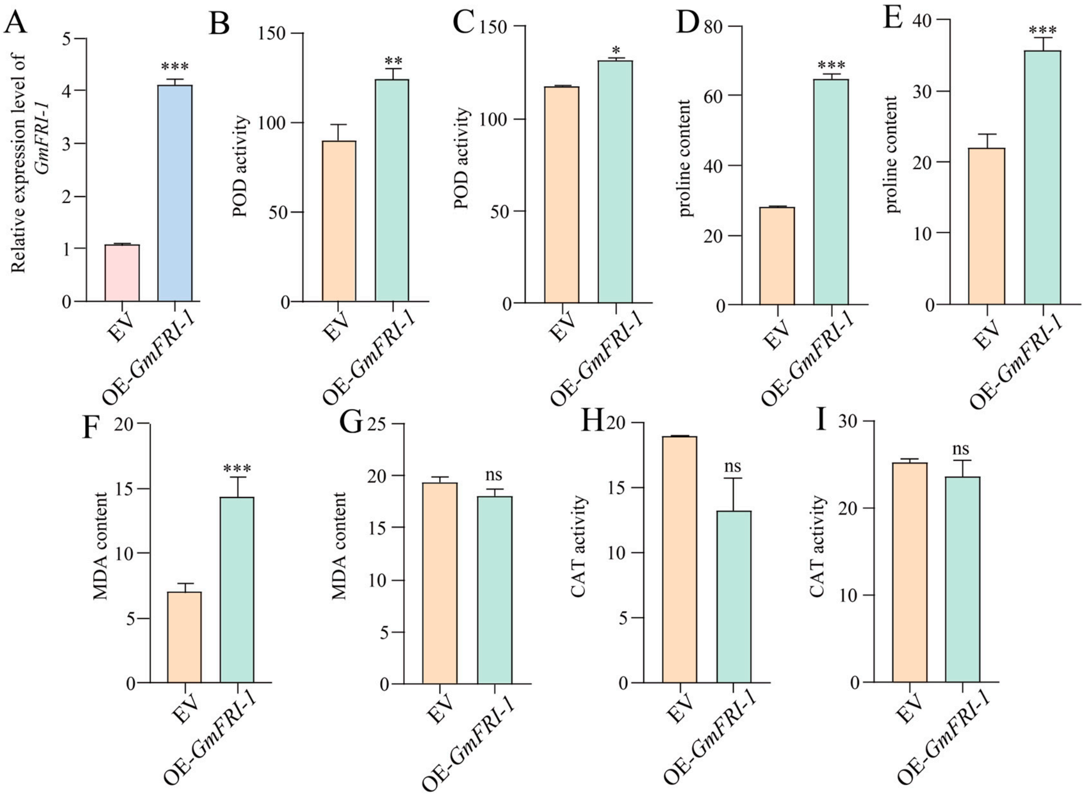

Supplementary Figure S3. Measurement of the physiological parameters associated with overexpressing *GmFRI-1* (OE-*GmFRI-1*) under both normal and low temperature circumstances, one day subsequent to the inoculation process. (**A**) Expression level of transgenic hairy roots harboring empty vector and 35S:*GmFRI-1*. The expression levels were normalized against the housekeeping gene of soybean *GmCYP2*. Student's *t*-test was performed (***p<0.001, n=20). (**B**) Contrast of the peroxidase (POD) activities between the OE-*GmFRI-1* samples and the control group under normal temperature conditions. (**C**) Comparison of the POD activities between the OE-*GmFRI-1* specimens and the control set under low temperature conditions. (**D**) Evaluation of the differences in proline content between the OE-*GmFRI-1* samples and the control under normal temperature conditions. (**E**) Analysis of the proline content disparities between the OE-*GmFRI-1* specimens and the control at low temperature. (**F**)Assessment of the malondialdehyde (MDA) content variations between the OE-*GmFRI-1* samples and the control under normal temperature conditions. (**G**) Comparison of the MDA content distinctions between the OE-*GmFRI-1* specimens and the control at low temperature. (**H**) Comparison of the catalase (CAT) activities between the OE-*GmFRI-1* samples and the control under normal temperature conditions. (**I**) Examination of the CAT activity differences between the OE-*GmFRI-1* specimens and the control under low temperature. *p<0.05, **p<0.01, and ***p<0.001; ns, no significance.

Table S3 Primers

Name	Sequence
qRT-*GmAPX1*-Forward	GGAGTTGGTTCAGACAGCCTTGG
qRT-*GmAPX1*-Reverse	CAGTGTCCATCCCAGCTTCATCAG
qRT-*GmAUX*-Forward	AGGGGACTGGATGATGGTTGGAG
qRT-*GmAUX*-Reverse	CGGTCAGCTCTTGTGATCTTCAGC
qRT-*GmDREB1A-2*-Forward	TAAGTGAGGTGAGGGAGCCCAAC
qRT-*GmDREB1A-2*-Reverse	CAGCGAAGTTGAGACAGGCATACC
qRT-*GmTCF1a*-Forward	CGGTGGCTGTGGATTCGCTATTG
qRT-*GmTCF1a*-Reverse	GGTTTCGGTTGGAAGAGGGAAAGG
qRT-*GmERD14*-Forward	TGAGGTTGAGGTCCAGGATCGTG
qRT-*GmERD14*-Reverse	GGCTGTGCTTGTTCTCTCCTTGG
qRT-*GmbZIP62*-Forward	GAGCCAAGGTGAAGTTAGCAGAGG
qRT-*GmbZIP62*-Reverse	CGCCGCAGATTAGTCGTGTTCAG
qRT-*GmFRI-1*-Forward	AGGACGTGGAGGCTTCATTG
qRT-*GmFRI-1*-Reverse	AACACTGGGAATCCCGAAGC
qRT-*GmFRI-2*-Forward	TCTAATGGTGGTAAAGGAGGG
qRT-*GmFRI-2*-Reverse	AGCAGCAAGGTTCTTACGGT
qRT-*GmFRI-3*-Forward	GAAGCCACAAAACCTCAACC
qRT-*GmFRI-3*-Reverse	TCAGCAACTCTAGCATAGGAAGC
qRT-*GmFRI-4*-Forward	AAAGCAGAGGAAGGTCACGA
qRT-*GmFRI-4*-Reverse	AGCAGCAAGGTTCTTACGGT
qRT-*GmFRI-5*-Forward	CCATGACCTTGAGACGGAGTT
qRT-*GmFRI-5*-Reverse	CACAGTCTTCCTTTCTGCGAGT
qRT-*GmFRI-6*-Forward	CTGGATTTACTGGAGGGGTTT
qRT-*GmFRI-6*-Reverse	TGCTTGGTTTGAGGGTTCA
qRT-*GmFRI-7*-Forward	TGGAAAACCTGAAGACACCTG
qRT-*GmFRI-7*-Reverse	AAGCGGAAGCAATAACAAGC
qRT-*GmFRI-8*-Forward	CGCTTGGAGGAAACAGATG
qRT-*GmFRI-8*-Reverse	AAGGGAACAGGAGGGAACTT
qRT-*GmFRI-9*-Forward	TCAGAACAAGCGAGGCAGTG
qRT-*GmFRI-9*-Reverse	GGTAACCAGACGGACCCAAA
qRT-*GmFRI-10*-Forward	ACAATTTGGGTCCGTCTGG
qRT-*GmFRI-10*-Reverse	TCCCCTGTAGGTGGTTTCAT
qRT-*GmFRI-11*-Forward	CTCATACTGTCCGTTGTCGC
qRT-*GmFRI-11*-Reverse	TCTTCCGCCTGGGTTACTT
qRT-*GmFRI-12*-Forward	ATAGGGCAAGGATTCAGGC
qRT-*GmFRI-12*-Reverse	TGTCATCATCACCCACCAAC
qRT-*GmFRI-13*-Forward	CGACCCATCATCGAATCAC
qRT-*GmFRI-13*-Reverse	CCCTACACCGTCCATTTTCT
qRT-*GmFRI-14*-Forward	CAGAAAACGCAGAGGGACA
qRT-*GmFRI-14*-Reverse	TAATCAACCTCCGCACCAC
qRT-*GmFRI-15*-Forward	CAGAGGGGCATTCAGGATAG

qRT-*GmFRI-15*-Reverse	GGTGGTTTCAGTGGCTTGTTC
qRT-*GmFRI-16*-Forward	CCATCCATCCCGCCTTATA
qRT-*GmFRI-16*-Reverse	TTGACCACGGTTGCCACTAG
qRT-*GmFRI-17*-Forward	TGGTGTTGAAGCGGAAGGA
qRT-*GmFRI-17*-Reverse	CTCTGCCAGTAGCGATGTTG
qRT-*GmENOD40*-Forward	TGGACAACACCCTCTAAACCA
qRT-*GmENOD40*-Reverse	GTGAGGGAGTGTGAGGAGTGA
qRT-*GmNIN*-Forward	CATCTTGAGCCTCTACCACC
qRT-*GmNIN*-Reverse	GCTTTGACTCTAAAAGTGCCGG
qRT-*GmNSP1*-Forward	GGTCTATAACTTTTGCTTCCAGC
qRT-*GmNSP1*-Reverse	CAGTGTCTTCGCCAAGAACTTG
qRT-*GmHAP2-1*-Forward	CACGCCATCTACATGCGAC
qRT-*GmHAP2-1*-Reverse	CAGTGTCTTCGCCAAGAACTTG
qRT-*GmHAP2-2*-Forward	GGAGTGCCTTAGGATCTCAACC
qRT-*GmHAP2-2*-Reverse	TACCGCTTGCTTACCGGCTG
qRT-*GmFRI-1*-RNAi-Forward	AGAGCACCTCTCTGTTGGT
qRT-*GmFRI-1*-RNAi-Reverse	ACACATGCTTCCCAGCTGAT
GmFRI-1- Forward	ATGGAAACCCCCGAAAACG
GmFRI-1- Reverse	CATATCAAAAACAGCATCAGCAAAG
GmFRI-1-207- Forward	GGGGACAAGTTTGTACAAAAAAGCA GGCTTCATGGAAACCCCCGAAAACG
GmFRI-1-207- Reverse	GGGGACCACTTTGTACAAGAAAGCT GGGTCCATATCAAAAACAGCATCAG CAAAG
